# *TERT* c.3150 G > C (p.K1050N): a founder Ashkenazi Jewish variant associated with telomere biology disorders

**DOI:** 10.1038/s41525-025-00501-8

**Published:** 2025-06-02

**Authors:** Kelvin César de Andrade, Emilia M. Pinto, Tianna Zhao, Logan P. Zeigler, Jung Kim, Neelam Giri, Jeremy S. Haley, Lisa J. McReynolds, Oscar Florez-Vargas, Aaron H. Phillips, Richard W. Kriwacki, Sherifa A. Akinniyi, Scott B. Cohen, Matthew R. Emerson, Diane T. Smelser, Gretchen M. Urban, Cintia Fridman, Gerard P. Zambetti, Tracy M. Bryan, David J. Carey, Christine Kim Garcia, Douglas R. Stewart, Sharon A. Savage

**Affiliations:** 1 Clinical Genetics Branch, Division of Cancer Epidemiology and Genetics, National Cancer Institute, National Institutes of Health, Bethesda, MD USA; 2 Department of Pathology, St. Jude Children’s Research Hospital, Memphis, TN USA; 3 Division of Cancer Epidemiology and Genetics, National Cancer Institute, National Institutes of Health, Bethesda, MD USA; 4 Cancer Genomics Research Laboratory, Frederick National Laboratory for Cancer Research, Frederick, MD USA; 5 Department of Genomic Health, Geisinger Clinic, Geisinger, Danville, PA USA; 6 Laboratory of Translational Genomics, Division of Cancer Epidemiology and Genetics, National Cancer Institute, National Institutes of Health, Bethesda, MD USA; 7 Department of Structural Biology, St. Jude Children’s Research Hospital, Memphis, TN USA; 8 Cell Biology Unit, Children’s Medical Research Institute, Faculty of Medicine and Health, University of Sydney, Westmead, NSW Australia; 9 Departamento de Medicina Legal, Bioética, Medicina do Trabalho e Medicina Física e Reabilitação, Faculdade de Medicina da Universidade de São Paulo, São Paulo, Brazil; 10 Division of Pulmonary and Critical Care Medicine, Columbia University, New York, NY USA

**Keywords:** Genetics, Haplotypes, Population genetics, Genetics research

## Abstract

Pathogenic germline variants in telomerase (*TERT*) cause telomere biology disorders (TBDs) and are associated with bone marrow failure, pulmonary fibrosis, and other complications. *TERT* c.3150 G > C (p.K1050N) is frequent in the Ashkenazi Jewish (ASH) population and has been identified in ASH families with TBDs. Whole-genome sequencing of 96 p.K1050N heterozygotes from the UK Biobank and *All of Us* databases revealed a shared haplotype block, supporting a founder effect. Analyses of 15 additional p.K1050N cases validated this haplotype and identified mitochondrial and Y-STR haplogroups consistent with ASH ancestry. Clinical assessments showed that p.K1050N contributes to TBD phenotypes and shortened telomeres, while population data suggest incomplete penetrance. p.K1050N reduces telomerase activity and processivity, and decreases PCNA expression and BrdU incorporation, impairing cell proliferation. Our findings establish *TERT* p.K1050N as an ASH founder variant associated with TBDs, underscoring the need for genetic screening and long-term clinical studies.

## Introduction

Telomere biology disorders (TBDs) are a spectrum of illnesses associated with high rates of bone marrow failure, pulmonary fibrosis, liver disease, cancer, and other complications. They are caused by pathogenic or likely pathogenic (P/LP) germline variants in genes essential in telomere maintenance resulting in short and/or dysfunctional telomeres^1^. Heterozygous P/LP variants in telomerase (encoded by *TERT*) are a common cause of adult-onset TBDs and rare biallelic variants in patients with childhood-onset disease have been reported^2^.

The *TERT* single nucleotide variant (SNV) c.3150 G > C (p.K1050N, rs373400596 – hereafter referred to as “p.K1050N”) was first identified in an Ashkenazi Jewish (ASH) family affected by TBDs^3^. Although rare in the general population—with a global minor allele frequency (MAF) of 0.006% in gnomAD (v4.1.0)^4^—p.K1050N is ~40-fold more frequent in the ASH population (MAF: 0.236%). This elevated frequency, coupled with its presence in additional TBD families, suggests that p.K1050N is a founder ASH variant associated with TBDs. In this study, we explored the genetic origin of the p.K1050N variant and its biological consequences.

## Results

### Defining the p.K1050N haplotype

**Fig. 1:**
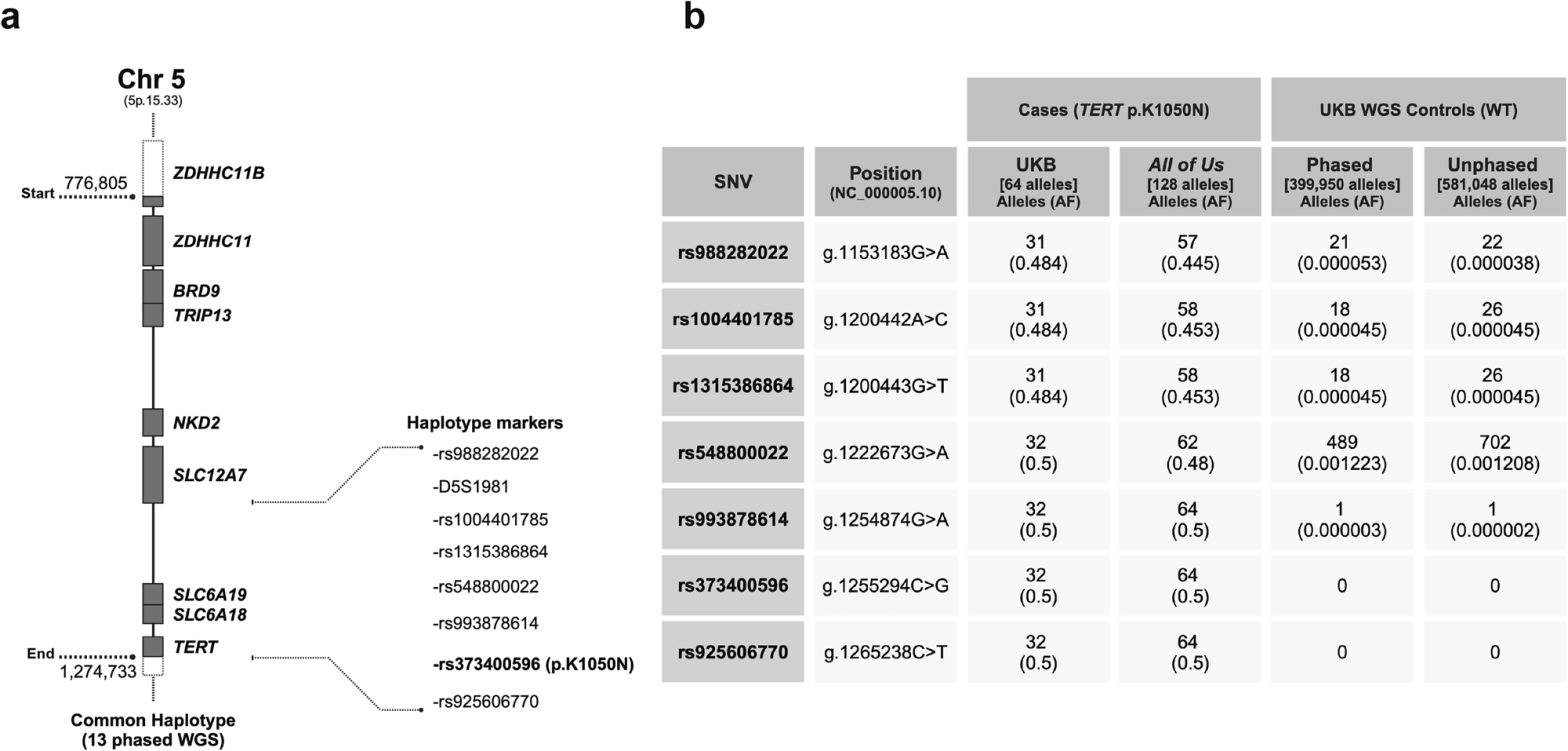
Founder haplotype structure *TERT* c.3150 G > C (p.K1050N). **a** Characterization of the region (chr5: 776,805–1,274,733; hg38) shared among all 13 UKB heterozygotes with phased WGS. **b** Allele frequency of the rare SNVs identified within the common haplotype in cases and controls. Abbreviations: SNV single-nucleotide variant, UKB UK Biobank, WGS whole-genome sequencing, WT wild-type, AF allele frequency. Created in BioRender. De Andrade, K. (2025) https://BioRender.com/n76x380.

Whole genome sequencing (WGS) data from the UK Biobank (UKB) and the *All of Us* databases were used to characterize the p.K1050N haplotype. Phased WGS data from 13 UKB p.K1050N heterozygotes revealed shared haplotype blocks of varying lengths. Notably, all cases shared a common 497,929 base pair region (chr5:776,805–1,274,733, hg38) containing six rare single nucleotide variants (SNVs) *in cis* with p.K1050N (Fig. 1a). This full haplotype, which also encompasses the polymorphic microsatellite marker D5S1981, co-segregates with the p.K1050N variant in the three families included in this study (NCI-258, F339, and F552). The six rare SNVs were also present in six independent clinical cohort cases (ID 4310355 and Geisinger, Table 1) and in 75 of 83 p.K1050N heterozygotes with unphased WGS data from the population sequencing databases (Fig. 1b). The remaining eight heterozygotes retained at least the two SNVs flanking p.K1050N, indicating partial haplotype conservation. Neither the full nor partial haplotypes were detected among the 199,975 phased WGS controls in the UKB database. Additionally, none of the 290,524 unphased WGS controls carried all six rare SNVs or the two flanking ones (Fig. 1b).

**Table 1:** Clinical characteristics of the individuals with the germline *TERT* c.3150 G > C (p.K1050N) variant

Data source	Family ID	Family member (ID)	Sex (*n*)	p.K1050N status	Clinical data (age at event/last follow-up)	Telomere length (%ile)	mtDNA haplogroup	Y-STR haplogroup	Reported ancestry (*n*)
Clinical cohorts									
NCI									
	NCI-258	Proband (NCI-258-1)	M	Hom.	Cytopenia (15, 26, 30)	<1st	H2a2a	G1bL830	ASH (4)
					Hypocellular bone marrow (26)				
					Avascular necrosis of hips (26)				
					Avascular necrosis of humoral heads (28, 29)				
		Father (NCI-258-3)*	M	Het.	Death due to pulmonary complications (79)	~10th	H3w	G1bL830	
		Mother (NCI-258-4)	F	Het.	No relevant conditions (69)	~10th	H2a2a	.	
		Sister (NCI-258-2)	F	Het.	No relevant conditions (36)	<1st	H2a2a	.	
Columbia university									
	F339	Proband (CKG1979)	F	Het.	Oral leukoplakia (65)	2nd-3rd	H1u2	.	NHW (3)
					Pulmonary fibrosis (65)				
		Son (CKG2327)	M	Het.	Liver failure (44)	<1st	H1u2	R1aM198	
					Peripheral neuropathy (44)				
					Atypical multiple sclerosis (44)				
		Daughter (CKG2325)	F	Het.	No relevant conditions (45)	<1st	H1u2	.	
	F552	Proband (CUMC0035)	M	Het.	Liver transplant (40)	4th–5th	K2a	R1aM198	NHW (2)
					Pulmonary fibrosis (53)				
					Lung transplant (55)				
		Brother (CUMC0542)	M	Het.	Pulmonary fibrosis (60)	<1st	K2a	R1aM198	
	.	Proband (4310355)	M	Het.	Pulmonary fibrosis (68)	1st-2nd	K1a9	J2a1	NHW (1)
Geisinger									
	.	GHS-1	F	Het.	Rectal Adenocarcinoma (47)	.	T2	.	EUR (5)
		GHS-2	F	Het.	Breast cancer (54)	.	HV	.	
		GHS-3*	F	Het.	COPD (63)	.	K1b1c	.	
		GHS-4	F	Het.	.	.	I2a1	.	
		GHS-5	M	Het.	.	.	H11a2a	R1bM269	
Population sequencing databases									
UK Biobank									
	.	UKB-1	F	Het.	Hodgkin lymphoma (16)	10th-50th	.	.	ASH (18) NFE (14)
					Breast cancer (51)		.	.	
		UKB-2	F	Het.	Breast cancer (60)	No data	.	.	
		UKB-3	M	Het.	Bladder cancer (65)	1st-10th	.	.	
		UKB-4*	F	Het.	COPD (73)	1st-10th	.	.	
		UKB-5:UKB-32	F (12) / M (16)	Het.	No relevant conditions (n = 24)	<10th (n = 5)	.	.	
					No diagnosis reported (n = 4)	>10th (n = 19)	.	.	
						No data (n = 4)	.	.	
All of us									
	.	AoU-1	M	Het.	Anemia (40s)	.	.	.	EUR (48) Remaining (16)
					Chronic nonalcoholic liver disease (40s)	.	.	.	
					Interstitial lung disease (40s)	.	.	.	
					Liver transplant (40s)				
					Primary thrombocytopenia (40s)	.	.	.	
					Leukopenia (50s)	.	.	.	
					Lung transplant (50s)	.	.	.	
		AoU-2	M	Het.	Anemia (60s)	.	.	.	
					Prostate cancer (60s)				
					Chronic nonalcoholic liver disease (70s)	.	.	.	
		AoU-3	F	Het.	Anemia (50s)	.	.	.	
					Thyroid cancer (50s)				
					Leukopenia (50s)	.	.	.	
		AoU-4*	F	Het.	Anemia (50s)	.	.	.	
					Colorectal cancer (50s)				
		AoU-5	F	Het.	Anemia (60s)	.	.	.	
					COPD (60s)				
		AoU-6*	M	Het.	Anemia (60s)	.	.	.	
					Tongue cancer (60s)				
		AoU-7*	M	Het.	Tongue cancer (60s)	.	.	.	
		AoU-8	M	Het.	Prostate cancer (50s)	.	.	.	
					Idiopathic pulmonary fibrosis (70s)	.	.	.	
		AoU-9*	M	Het.	Colorectal cancer (60s)	.	.	.	
					Leukoplakia of oral mucosa (70s)	.	.	.	
		AoU-10	F	Het.	Colorectal cancer (70s)	.	.	.	
		AoU-11*	F	Het.	COPD (50s)	.	.	.	
					Anemia (60s)				
		AoU-12	F	Het.	Chronic lymphocytic leukemia (70s)	.	.	.	
					Chronic myeloid leukemia (70s)	.	.	.	
		AoU-13	M	Het.	Chronic nonalcoholic liver disease (50s)	.	.	.	
					Anemia (60s)				
		AoU-14*	F	Het.	Anemia (70s)	.	.	.	
					Leukopenia (70s)				
		AoU-15	M	Het.	Crohn’s disease (20s)	.	.	.	
		AoU-16*	M	Het.	Crohn’s disease (50s)	.	.	.	
		AoU-17:AoU-64	F (22) / M (26)	Het.	No relevant conditions (n = 31)	.	.	.	
					No EHR available (n = 17)	.	.	.	

Family NCI-258 tested negative for *RTEL1* c.3791 G > A (p.R1264H) and for additional variants in *TERC* and *MPL*. Family F339 tested negative for *RTEL1* c.3791 G > A (p.R1264H) and for variants in *TERC*. Family F552 and ID 4310355 were analyzed for 28 TBD-associated genes (listed in Methods). Individuals from Geisinger, UKB, and *All of Us* were only evaluated for the co-occurrence with the *RTEL1* c.3791 G > A (p.R1264H) variant. Members of family F552 and AoU-1 are also heterozygous for the ASH founder germline *RTEL1* c.3791 G > A (p.R1264H) variant. To protect confidentiality, ages at diagnoses for individuals in the *All of Us* database were masked. Telomere lengths were measured using flow FISH for samples from the NCI and qPCR for samples from Columbia University and the UKB. *NCI* National Cancer Institute, *M* male, *F* female, *Hom* homozygote, *Het* heterozygote, *COPD* chronic obstructive pulmonary disease, *ASH* Ashkenazi Jewish, *NHW* non-Hispanic White, *EUR* European, *NFE* non-Finnish European, *EHR* electronic health record, *%ile* percentile. * Previous or current smokers

### Ancestry analysis

Mitochondrial DNA (mtDNA) analysis showed that the 15 individuals with the *TERT* p.K1050N variant from the clinical cohorts belonged to either macrohaplogroup R (which includes haplogroups H, HV, K and T) or macrohaplogroup N (which includes haplogroup I) (Table 1). Y-STR analysis identified macrohaplogroups J, R, and G. Notably, male family members from two unrelated families (F339 and F552) share the same Y-STR subclade, R1aM198 (Table 1).

### Clinical manifestations and telomere length

Among individuals in the clinical cohorts (Table 1), the earliest TBD-associated diagnoses were observed in the homozygous proband (NCI-258-1), whereas five heterozygotes (F339, F552, and ID 4310355) exhibited a later onset of TBD-associated conditions. All individuals with available clinical telomere length (TL) measurements (n = 10) had TL near or below the 10^th^ percentile compared with age-matched populations (Table 1).

Among the 96 p.K1050N heterozygotes identified in the population sequencing databases, one male individual (AoU-1), who also carried the *RTEL1* c.3791 G > A founder ASH variant, developed TBD-related diagnoses in his 40s. Other participants reported various cancers and clinical symptoms potentially associated with TBDs (Table 1). Unfortunately, electronic health records (EHR) were unavailable for 17 out of 64 *All of Us* participants. In the UKB, four out of 32 heterozygotes had no diagnoses reported in the data fields assessed (Methods). TL varied widely among UKB heterozygotes, with a notable tendency toward shorter TL compared with the median of the control population. Among 27 individuals with available TL data, seven had TL below the 10th percentile for their age (Table 1, Supplementary Fig. 1).

### Structural and functional impact of the TERT p.K1050N variant

**Fig. 2:**
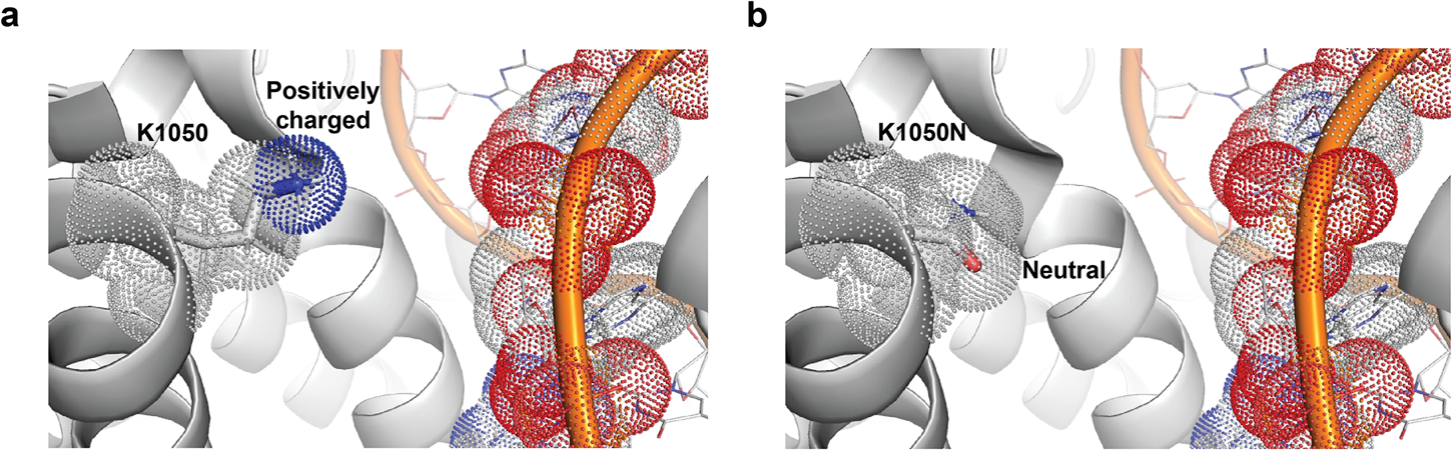
Impact of *TERT* p.K1050N on telomerase structure. **a** The location of residue position 1050 in the cryo-EM structure of telomerase in complex with the RNA:DNA hybrid is shown (PDB code 5BG9)^44^. Residue 1050 and the RNA strand are shown in a surface representation with the experimental structure of K1050. **b** Lowest energy rotamer of asparagine as determined by PyMol^40^.

**Fig. 3:**
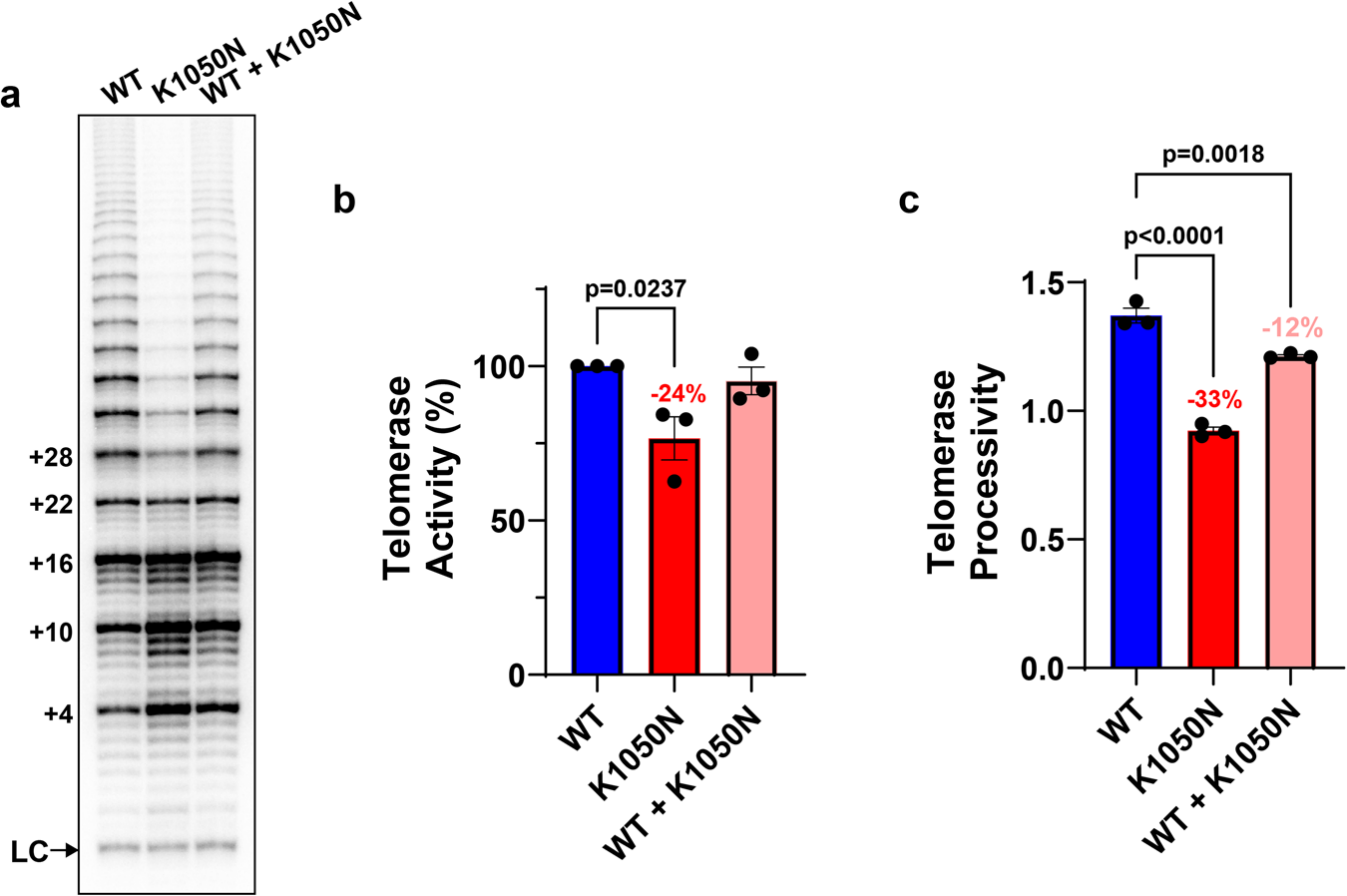
Impact of *TERT* p.K1050N on telomerase activity and processivity. **a** Direct telomerase activity assay of immunopurified telomerase expressed in HEK293T cells, using plasmid encoding WT TERT, the p.K1050N variant, or an equimolar mixture of WT and p.K1050N. Each prominent band represents the addition of a TTAGGG repeat to the DNA substrate, with the number of nucleotides added indicated on the left. LC: labelled, biotinylated 30nt oligonucleotide recovery and loading control. **b** Specific activity of K1050N and K1050N + WT, compared to WT telomerase. **c** Processivity of WT, K1050N and K1050N + WT, relative to WT telomerase. Processivity is defined as the average number of DNA repeats synthesized before the enzyme dissociation from DNA. Data points represent independent immunopurified telomerase samples, with error bars indicating mean + SEM (*n* = 3). Abbreviations**:** WT wild-type. Plots created with GraphPad Prism version 10.0.0 (www.graphpad.com).

Residue p.K1050 is located in the C-terminal extension (CTE) domain of TERT. FoldX calculations predicted that substitution of lysine with asparagine at this position would be tolerated (Fig. 2). Our analysis demonstrates that p.K1050N does not impact hTERT expression or stability, or hTR association with hTERT (Supplementary Fig. 2). We next characterized the functional impact of p.K1050N on telomerase activity and repeat addition processivity. Compared with wild-type (WT) levels, the p.K1050N variant reduced telomerase activity (Fig. 3a) and processivity (Fig. 3b) by 24% and 33%, respectively. When co-expressed with WT telomerase to mimic a heterozygous state, the reduction in activity was no longer statistically significant (Fig. 3b); however, a significant 12% decrease in processivity remained (Fig. 3c). These reductions were comparable to or greater than those observed for the *TERT* p.V694M variant (Supplementary Fig. 3). Moreover, the p.K1050N variant reduced both PCNA expression and bromodeoxyuridine (BrdU) incorporation in primary patient-derived fibroblasts, particularly in the homozygous state, indicating impaired cell proliferation (Supplementary Fig. 4).

## Discussion

In this study, we present evidence that *TERT* c.3150 G > C (p.K1050N) is a founder variant in the ASH population. The variant’s pathogenic potential is demonstrated by impaired telomerase activity and processivity, as well as reduced cell proliferation, highlighting its clinical relevance in TBDs.

The founder effect in the ASH population is supported by the identification of a haplotype block shared exclusively among individuals with the p.K1050N variant and by our mtDNA and Y-STR findings. Both mtDNA macrohaplogroups identified (R and N) include haplogroups that are commonly observed in ASH maternal lineages throughout Europe^5^. For instance, haplogroup K is the most prevalent among the ASH population, found in approximately 32% of individuals^6^, compared with only ~6% in Near Eastern and European non-Jewish populations^7^. The Y-STR macrohaplogroups J, R, and G account for approximately 38%, 19%, and 10% of all ASH paternal lineages in Europe, respectively^8^. The presence of the same Y-STR subclade in unrelated families reinforces a shared genetic origin. The high frequency of the p.K1050N variant in the ASH population, along with these mtDNA and Y-STR findings, strongly supports p.K1050N as a distinct founder variant within this population.

Data from our clinical databases demonstrate that the p.K1050N variant can lead to short telomeres and to the subsequent development of TBD-associated phenotypes, consistent with previous reports^3,9^. Although only a single p.K1050N homozygote was identified, our results suggest that the homozygous state may result in earlier or more aggressive phenotypes. Members of family F552 were also heterozygous for the *RTEL1* c.3791 G > A (p.R1264H) variant, previously identified as a recessive ASH founder variant in TBD families^10^. While the clinical and functional consequences of *RTEL1* p.R1264H heterozygosity have not been well studied, it is possible that both variants may have contributed to the clinical manifestations observed in family F552. Longitudinal studies of individuals with *TERT* p.K1050N, particularly homozygotes, with or without *RTEL1* p.R1264H are essential to better characterize the phenotypic spectrum and refine risk estimates.

Data from the population sequencing databases demonstrated variability in clinical presentation of p.K1050N heterozygotes, suggesting that disease expression may be influenced by additional genetic modifiers or other risk factors, such as aging and smoking. There was no clear association between TL and the development of TBD-associated conditions in the UKB, likely due to the small sample size. Importantly, while qPCR is a cost-effective approach for TL measurement in large populations, it is not sensitive enough for clinical diagnoses of individual patients^11,12^. The low prevalence of TBD manifestations in the population sequencing databases could be attributed to the lack of complete clinical data for a subset of individuals, and database-related intrinsic survival biases, exacerbated by the “healthy volunteer effect” documented in the UKB^13^. Consequently, the clinical findings reported in these databases likely underestimate the full TBD phenotypic spectrum associated with the p.K1050N variant.

A previously identified TBD-associated lysine-to-glutamate substitution (p.K1050E) impaired the CTE domain’s nucleic acid binding affinity, reducing both telomerase activity and processivity by ~2-fold^14,15^, and reducing binding of the CTE domain to an RNA:DNA hybrid in vitro^14^. The p.K1050N mutation likely disrupts interactions with the nucleic acid phosphate backbone due to the absence of a positive charge in the asparagine side chain. While our computational modeling suggests that p.K1050N may impair these interactions, direct experimental evidence for p.K1050N requires further investigation.

The p.K1050N variant reduced telomerase activity and processivity to levels similar to those of the *TERT* p.V694M variant, which served as a benchmark for P/LP-associated deficits in our functional assay^16^. Of note, in vitro telomerase activity for variants with processivity defects can be influenced by enzyme turnover and may not accurately reflect in vivo activity^17^, as observed with another variant affecting the same codon, *TERT* p.K1050E^15^. Nonetheless, defects in telomerase processivity have been shown to impact telomere length maintenance in vivo^17,18^. The impact of the p.K1050N variant was further substantiated by its reductions in PCNA expression and BrdU incorporation in primary patient-derived fibroblasts, particularly in the homozygous state. These results provide evidence that p.K1050N impairs telomerase function and cellular processes critical for telomere maintenance, underscoring the pathogenic nature of the p.K1050N variant and its significant role in TBDs.

Current variant classification guidelines from the American College of Medical Genomics and the Association for Molecular Pathology (ACMG/AMP)^19^ do not fully address curation of variants with incomplete penetrance or founder effects. Based on adapted criteria for TBD-associated genes, the p.K1050N variant was recently classified as likely pathogenic^1^. Our data corroborate this classification and provide additional clinical, *in-silico*, and functional evidence linking it to relevant TBD phenotypes. Therefore, we recommend genetic screening in at-risk populations and following TBD clinical surveillance guidelines^20^ for individuals with the p.K1050N variant.

Our study identifies *TERT* c.3150 G > C (p.K1050N) as a founder variant in the ASH population. The variant’s pathogenic potential is supported by reduced telomerase activity, processivity, and cell proliferation, along with shortened telomeres and association with typical TBD phenotypes. Longitudinal studies are encouraged to fully characterize the variant’s phenotypic spectrum and refine clinical management strategies.

## Methods

### Subjects (clinical cohorts)

Members of the NCI-258 family (n = 4, one homozygote, three heterozygotes), self-reported as Ashkenazi Jewish, were enrolled in the National Institutes of Health (NIH) institutional review board-approved Cancer in Inherited Bone Marrow Failure Syndromes (IBMFS) study (clinicaltrials.gov, Identifier: NCT00027274, registration date: November 30^th^, 2001)^21^ sponsored by the National Cancer Institute (NCI). Each individual underwent comprehensive evaluations at the NIH Clinical Center, including diagnostic imaging and blood tests per study protocol. The *TERT* c.3150 G > C (p.K1050N) variant was identified via Sanger sequencing at the NCI’s Cancer Genomic Research Laboratory and confirmed in a Clinical Laboratory Improvement Amendments (CLIA)-certified facility. Variants in *TERC, MPL*, and *RTEL1* p.R1264H were not identified in family NCI-258 via Sanger sequencing.

Members of families F339 (n = 3 heterozygotes) and F552 (n = 2 heterozygotes), and one unrelated heterozygote (ID 4310355), participated in the IRB-AAAS0753 and IRB-AAAR1916 studies at Columbia University Medical Center. All individuals self-reported Non-Hispanic White ancestry. The proband of family F339 had previously been identified with the *TERT* c.3150 G > C variant in an interstitial lung disease study^22^. Variants in *TERT*, *TERC*, and *RTEL1* p.R1264H were not identified in family F339 via Sanger sequencing.

Whole-genome sequencing (WGS)^23^ was performed on the proband of family F552 and ID 4310355, assessing 28 telomere biology disorder (TBD)-associated genes (*ACD, ACYP2, CBX3, CTC1, DKC1, GAR1, NAF1, NHP2, NOP10, OBFC1, PARN, POT1, RAP1, RPA1, RTEL1, STN1, TEN1, TEP1, TERC, TERF1, TERF2, TERF2IP, TERT, TINF2, TNKS, WRAP53, ZCCHC8*, and *ZNF208*). In addition to the *TERT* c.3150 G > C variant, the *RTEL1* c.3791 G > A (p.R1264H) was also identified in the family F552 proband and later confirmed in his brother via Sanger sequencing. No other TBD-related variants were identified in ID 4310355.

Five p.K1050N heterozygotes (ages at recruitment: 20, 61, 65, 67, and 85 years) were identified in the Geisinger DiscovEHR cohort using whole-exome sequencing (WES)^24^. None of them carried the *RTEL1* c.3791 G > A variant. These individuals were determined to be of European ancestry through principal component (PC) analysis of WES data^25^ (Supplementary Fig. 5). Electronic health records (EHR) and cancer registry data were retrieved, and a thorough review of clinical charts was conducted to capture all relevant diagnoses affecting the blood, gastrointestinal tract, lung, liver, or skin.

Genomic DNA from all individuals was available for haplotype and ancestry analyses. Informed consent was obtained from all participants. This study was conducted in accordance with all relevant ethical regulations, including the Declaration of Helsinki^26^.

### Population sequencing databases

UK Biobank (UKB) WGS data^27–29^ were accessed via the UKB DNA Nexus Research Platform. WGS was available for 490,499 individuals (data field 23374)^30^; with a subset of 199,975 individuals also having phased WGS data (data field 20279)^31^. We identified 32 *TERT* c.3150 G > C heterozygotes (median age at recruitment: 55 years, range 40–68 years), including 13 with also phased WGS data. No co-occurrence with the *RTEL1* c.3791 G > A variant was identified. Disease-related data fields queried included 40001 (underlying cause of death), 40006 (type of cancer ICD10), 40008 (age at cancer diagnosis), 40011 (histology of cancer tumor), 40012 (behavior of cancer tumor), 40013 (type of cancer ICD9), 41270 (diagnoses ICD10), 41271 (diagnoses ICD9), and 41280 (Date of first in-patient diagnosis - ICD10). Phenotype data were extracted in July 2024. Ancestry information from the UKB allele variant browser, classified 18 heterozygotes as ASH and 14 as Non-Finnish European. Participants who withdrew from the UKB cohort (n = 351; as of July 2024) were excluded from all analyses.

Additionally, WGS was available for 414,822 individuals from the *All of Us* Research Program database (version 8)^32,33^. We identified 64 *TERT* c.3150 G > C heterozygotes (35 males and 29 females), including 48 of European ancestry and 16 with ancestry categorized as “Remaining”, according to the *All of Us* public data browser. Median age at recruitment was 66.2 years (range 21.6–92.3 years). Two individuals were also heterozygous for the *RTEL1* c.3791 G > A variant. Clinical and genetic data for these individuals were obtained through approved access to the Controlled Tier (CDRv8), in accordance with the program’s data use policies. All analyses adhered to the ethical and regulatory standards set by the *All of Us* Research Program. Participants provided informed consent to use their de-identified genetic and clinical information for research purposes. We received an exception to the Data and Statistics Dissemination Policy from the *All of Us* Resource Access Board.

For both databases, phenotype analyses included primary malignant neoplasms (excluding non-melanoma skin cancer and uterine cervix cancer) and TBD-related diseases affecting the blood, gastrointestinal tract, lung, liver, or skin.

### Genetic profiling

Short tandem repeat (STR) profiling was performed using the PowerPlex Fusion STR system (Promega, Madison, WI, USA), which simultaneously amplifies 24 STR loci from DNA. This system utilizes five fluorescent dyes attached to primers to label the amplified PCR fragments. The dye-labeled PCR products were separated and detected with a 3500XL Genetic Analyzer with POP-7 polymer. Data analysis was conducted using GeneMapper 6.0 (ThermoFisher Scientific).

### Relatedness evaluation

STR profiling found no evidence of relatedness among participants from the clinical cohorts (data not shown to preserve participants’ confidentiality). In Geisinger DiscovEHR, identity-by-descent analysis (PLINK 1.9^34^) further confirmed no relatedness, up to the third degree, based on pairwise pi-hat values. Kinship coefficients in the UKB^28^ and *All of Us*^33^ identified two pairs of p.K1050N heterozygotes in each database with kinship scores above 0.1, indicating first- or second-degree relationships.

### Microsatellites

Fluorescent-labelled PCR products for four polymorphic microsatellite markers (D5S392, D5S1981, D5S2005, and D5S678; Supplementary Table 1) located within chr5:302,025–1,365,914 (hg38) were genotyped using the ABI 3500 sequencer (ThermoFisher Scientific). Fragment sizes were assigned using GeneMapper v6.0 (ThermoFisher Scientific).

### Defining a founder haplotype

Haplotype profiles of chromosome 5p, covering the region 13,600–10,400,000 (GRCh38/hg38), were analyzed in 13 *TERT* c.3150 G > C heterozygotes from the UKB with phased WGS. The smallest shared haplotype on chromosome 5p, encompassing c.3150 G > C, was identified across all these individuals. To define a founder haplotype, we filtered this region for single nucleotide variants (SNVs) with a global minor allele frequency (MAF) < 1% in gnomAD v.4.1 non-UKB genomes^4^, resulting in six rare SNVs. These SNVs were subsequently genotyped by Sanger sequencing (Supplementary Table 1) in all 15 individuals. Furthermore, these SNVs were investigated in the unphased WGS data from the UKB and *All of Us* population databases.

Haplotypes were determined by segregation analysis of SNVs and polymorphic markers in the NCI-258, F339, and F552 families, and were inferred in the independent heterozygotes without additional family members included in the study (ID 4310355 and Geisinger). Phased WGS data from 199,975 UKB controls (individuals without *TERT* c.3150 G > C) were evaluated to assess the frequency of the haplotype containing the alternate alleles for the six rare SNVs *in cis* with the wild-type (WT) allele in the chr5:1,255,294 position (A-C-T-A-A-C-T; WT allele underlined). Unphased WGS data from the remaining 290,514 UKB controls were analyzed to further assess the allele frequency of the six SNVs within the UKB database.

### Mitochondrial DNA control region

The nucleotide sequences of the mitochondrial DNA (mtDNA) hypervariable segments 1 (HVI; positions 16024 and 16365), 2 (HV2; positions 73 and 340), and 3 (HV3, positions 438 to 574) within the control region of mtDNA were determined in all individuals from the clinical cohorts. The mtDNA control region was amplified using the primers L15781 (5’-CCCTTTTACCATCATTGGACA-3’) and H727 (5’-AGGGTGAACTCACTGGAACG-3’). Sequencing of the amplified segments was carried out with primers L15781, H16478, L109, H408, and H727, as previously described^35,36^. The resulting sequences were compared to the Homo sapiens mitochondrial genome (NCBI reference sequence: NC_012920.1), also known as the revised Cambridge Reference Sequence (rCRS). mtDNA haplogroups were identified using Haplogrep software (https://haplogrep.i-med.ac.at/), EMPOP (https://empop.online/), and visual inspection.

### Y-chromosome markers

Twenty-three Y-STRs were analyzed for five males from clinical cohorts using the PowerPlex Y23 System (Promega), following the manufacturer’s instructions. The alleles were separated and detected with an ABI 3500 sequencer (ThermoFisher Scientific) and the fragment sizes were assigned using GeneMapper v 6.0 (ThermoFisher Scientific). The alleles were named according to the number of repeated units, as per the sequenced allelic ladder and the recommendations of the International Society for Forensic Genetics^37^. Y-STRs haplogroup were classified using the Haplogroup Predictor program FTDNA 2.0 (http://www.hprg.com/hapest5/index.html) and the Y-chromosome Haplotype Reference Database (https://yhrd.org/search).

### Telomere length measurement

Lymphocyte telomere lengths were measured by flow FISH at Repeat Diagnostics, Inc. (Vancouver, BC, Canada) for the NCI-258 family members, with percentiles calculated based on clinical testing parameters^38^. Leukocyte telomere lengths (LTL) were measured by qPCR for members of families F339, F552, and ID 4310355, as previously described^22^. Telomere length measurements were not determined for Geisinger participants (n = 5).

In the UKB, LTL was determined by qPCR (data field 22192, Z-adjusted T/S log) and was available for 27 out of 32 p.K1050N heterozygotes. Percentiles were calculated based on LTL data from UK controls (n = 460,541) which excluded participants (n = 12,128) with rare coding germline *TERT* variants (MAF < 1% in gnomAD v4.1.0 non-UKB). Telomere length data were not available in the *All of Us* database.

### Computational modelling of structural impact

Structural effects of lysine to asparagine (p.K1050N) in the TERT protein were predicted using FoldX^39^, using the reported crystal structure^14^, and visualized by PyMOL (https://pymol.org/2/)^40^.

### Telomerase activity and processivity (direct assay)

The *TERT* c.3150 G > C variant was introduced by site-directed mutagenesis into a plasmid encoding FLAG-tagged hTERT under a CMV promoter. This plasmid was transfected into HEK293T cells along with a plasmid encoding *TERC* (the RNA component of telomerase, hTR) and *DKC1* (dyskerin)^41^. Wild-type (WT) hTERT was transfected as a control, and a third transfection included equimolar amounts of WT and c.3150 G > C, to represent the heterozygous state. Telomerase was immunopurified using anti-FLAG M2 antibody affinity gel (Sigma), and hTERT levels were quantified by Western blot using an anti-FLAG antibody^41^ (Supplementary Table 2). Binding of hTERT to hTR (i.e. the potential impact of each variant on enzyme assembly) was assessed by measuring the amount of hTR recovered after immunopurification (IP) of hTERT by northern dot-blot^41^. The relative amounts of recovered hTR were normalized to the relative amounts of recovered hTERT (i.e. [% hTR relative WT]/[% hTERT relative to WT] in the IP eluate was calculated), and the result was also normalized to the initial hTR levels in the cell lysate to control for differences in expression from the plasmid encoding hTR. Equal volumes of immunopurified telomerase were used in direct telomerase activity assays, involving extension of a telomeric primer with radiolabeled nucleotides, followed by electrophoresis on a high-resolution acrylamide gel^41^.

Telomerase activity (the enzyme’s ability to add nucleotides to a DNA molecule) was quantified by analyzing the sample lanes and normalizing the results to the amount of hTR recovered. The activity was expressed as a percentage of WT telomerase activity. Processivity (the ability of telomerase to continuously extend a single DNA molecule) was quantified as previously described^42^.

A control panel consisting of three *TERT* variants previously classified^16^ as pathogenic or likely pathogenic (P/LP) - p.K570N, p.R865H, and p.V694M—and four *TERT* variants classified as benign or likely benign (B/LB)—p.A279T, p.A1062T, p.S191T, and p.E280K - was included in the direct telomerase activity assay.

### PCNA protein expression

Primary fibroblasts from NCI-258 family members were used to assess PCNA protein expression, a marker of cell proliferation^43^. Primary fibroblasts from NCI-258 family members and paired controls (Coriell Cell Repository) (Supplementary Table 3) were cultured in AmnioMAXTM-II Complete Medium (Thermo Fisher Scientific) supplemented with 1% Penicillin-Streptomycin. All cells were maintained in a 5% CO_2_ incubator at 37°C. Cultured fibroblasts were harvested and lysed in RIPA buffer (Sigma) supplemented with a dual protease/phosphatase inhibitor cocktail (Thermo Fisher Scientific) for 30 min on ice. The extracted proteins were separated on 4–12% Bis-Tris gels (Thermo Fisher Scientific) and transferred onto polyvinylidene difluoride (PVDF) membranes (MilliporeSigma). The membranes were incubated overnight at 4 °C with the indicated primary antibodies, followed by near-infrared fluorescent conjugated secondary antibodies (Supplementary Table 2). Protein expression was visualized by Odyssey DLx near-infrared imager and quantified with the Empiria Studio Software (LICORbio).

### BrdU cell proliferation assay

Primary fibroblasts from NCI-258 family members and their matched controls were seeded in 96-well plates (CELLTREAT) at a density of 5000 cells/well in 100 µL/well of AmnioMAXTM-II Complete Medium, with 4 replicates/sample. The control wells contained only medium without cells. After 24 h of culture, BrdU reagent was added in the cell media at 1:500 and incubated for an additional 24 h. BrdU cell proliferation assay (Abcam) was conducted according to the manufacturer’s instructions. Absorbance was measured at dual wavelengths of 450/560 nm using a GloMax Explorer plate reader (Promega). Sample absorbance was calculated by subtracting the absorbance of the blank controls.

## Supplementary information

Supplementary materials

## Data Availability

UK Biobank data are available to approved researchers upon application. Access requires completion of a data access application, which includes a research proposal and approval from the UK Biobank's Ethics and Governance Council. Due to privacy and ethical considerations, data are not publicly available. Access to MyCode data is restricted and requires approval from the Geisinger Institutional Review Board (IRB) and adherence to the MyCode data access policies. These data are not publicly available due to privacy and ethical restrictions. Researchers interested in accessing the MyCode data should submit a formal request through the MyCode Research Portal. Access to “*All of Us*” data is available through the *All of Us* Research Hub, where researchers can apply for access to participant data. Data are available to approved researchers who have completed the necessary training and data use agreements. Due to the sensitive nature of the data, access is governed by the *All of Us* Institutional Review Board (IRB) and Data and Research Center (DRC), and the National Cancer Institute does not have authority to share any of these data. Authors have received an exception to the Data and Statistics Dissemination Policy from the *All of Us* Resource Access Board.

## Code availability

We utilized publicly available bioinformatics tools and libraries, including BCFtools and UNIX commands for data processing. Python libraries such as Pandas for data manipulation, NumPy for numerical operations, Matplotlib and Seaborn for data visualization. All these tools are freely accessible, and detailed documentation can be found through their respective repositories or platforms. Additionally, the UK Biobank “Swiss Army knife” tool was employed for general data handling within the Research Hub. No new code was developed for this study.
