# Supplementary material for: TERT c.3150 G > C (p.K1050N): a founder Ashkenazi Jewish variant associated with telomere biology disorders
Source: NPJ Genom Med. 2025 Jun 2;10:46. doi: 10.1038/s41525-025-00501-8 (PMC12130525; doi:10.1038/s41525-025-00501-8)

Supplementary Table 1. Primers for single nucleotide variant and polymorphic marker genotyping**.**

Marker location (hg38)	ID	Forward primer	Reverse primer
chr5:1153183:G-A	rs988282022	TCATTCAGCTGATGGTTGGC	CACCCACCTCATCTCCTCTT
chr5:1200442:A-C	rs1004401785	CCAGGAGGCTAGGGAGAAAG	TGTGTCTCCAAAGCCAGAGT
chr5:1200443:G-T	rs1315386864	CCAGGAGGCTAGGGAGAAAG	TGTGTCTCCAAAGCCAGAGT
chr5:1222673:G-A	rs548800022	GTATGCCTGTGTGACGTGTG	GACAAACACAGAGGGCAGC
chr5:1254874:G-A	rs993878614	TGTTAAGCTCAGGACCGCAG	ATCCTTCCCACCTTGCTCTG
chr5:1265238:C-T	rs925606770	AAGGGGAGGGTTCTGAGTTC	GCAGCCATGTAACAGGAAGG
chr5:302025-302151	D5S392	GCTATTCCCACAAAGGCA	GGCGGATCATTGAGTGC
chr5:1154299-1155062	D5S1981	CCTGTACCAATCCATGC	GAGCCATGTGAGTGTCC
chr5:1341960-1342197	D5S2005	CCTCAGGTGGGTTATTGAC	CCCAGGGCTTTACGAGT
chr5:1365615-1365914	D5S678	CATGAGTCCCGTGACTTTGT	GGCAGGAGAATTGCATGA

Supplementary Table 2. Antibodies used in this study

Antibody	Supplier	Catalog	Dilution
PCNA	Cell Signaling Technology	13110T	1/1,000
GAPDH	Cell Signaling Technology	97166S	1/1,000
Anti-FLAG M2	Sigma-Aldrich	F3165	1/5,000
Tubulin	Invitrogen	PA1-38814	1/2,500
IRDye 800CW Goat anti-Rabbit IgG Secondary Antibody	LICORbio	925-32211	1/20,000
IRDye 680RD Goat anti-Mouse IgG Secondary Antibody	LICORbio	925-68070	1/20,000

Supplementary Table 3. Information on primary fibroblasts derived from controls and individuals with the *TERT* c.3150G>C (p.K1050N) variant.

Samples	ID	*TERT* c.3150G>C	Age at Sample Collection	Sex	Passage Number (PCNA)	Passage number (BrdU)
Ctl1	AG16104	.	55	Female	10	·
Ctl2	GM03658 B	.	68	Male	9	10
Ctl3	GM07545 A	.	22	Female	6	·
Ctl4	GM07753 B	.	17	Male	9	10
TC1	NCI-258-3	Heterozygote (father)	69.4	Male	9	10
TC2	NCI-258-4	Heterozygote (mother)	54.6	Female	9	·
TC3	NCI-258-2	Heterozygote (sibling)	21.2	Female	9	·
TC4	NCI-258-1	Homozygote (proband)	15.9	Male	8	9

Abbreviations: Ctl, control; TC: *TERT* cases

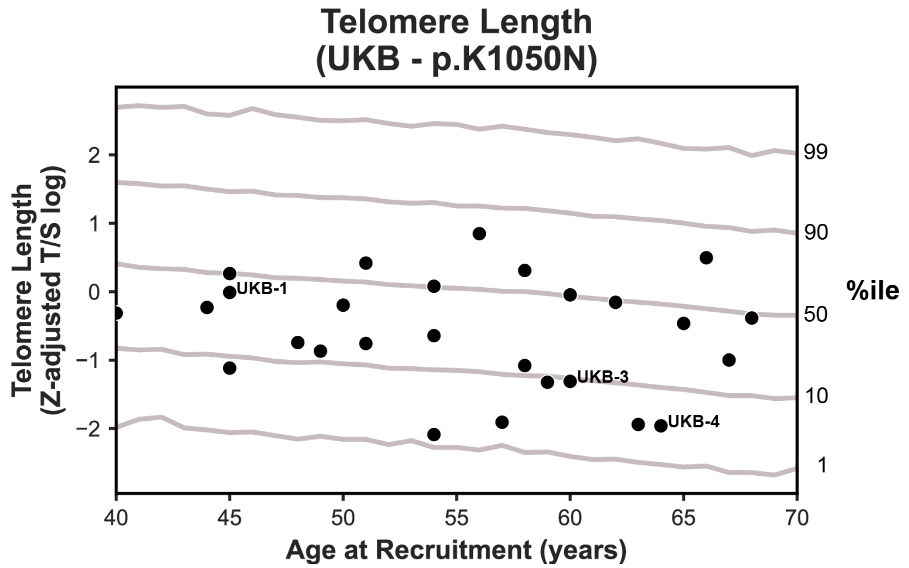

Supplementary Figure 1. Telomere length for 27 *TERT* c.3150G>C heterozygotes from the UKB database. Individuals with relevant phenotypes listed in Table 1 are labeled, except for UKB-2, who lacked LTL data. Percentiles were calculated using UKB controls (n=460,541). See methods for details. Plot created using the Python libraries Matplotlib (https://matplotlib.org) and Seaborn (https://seaborn.pydata.org).

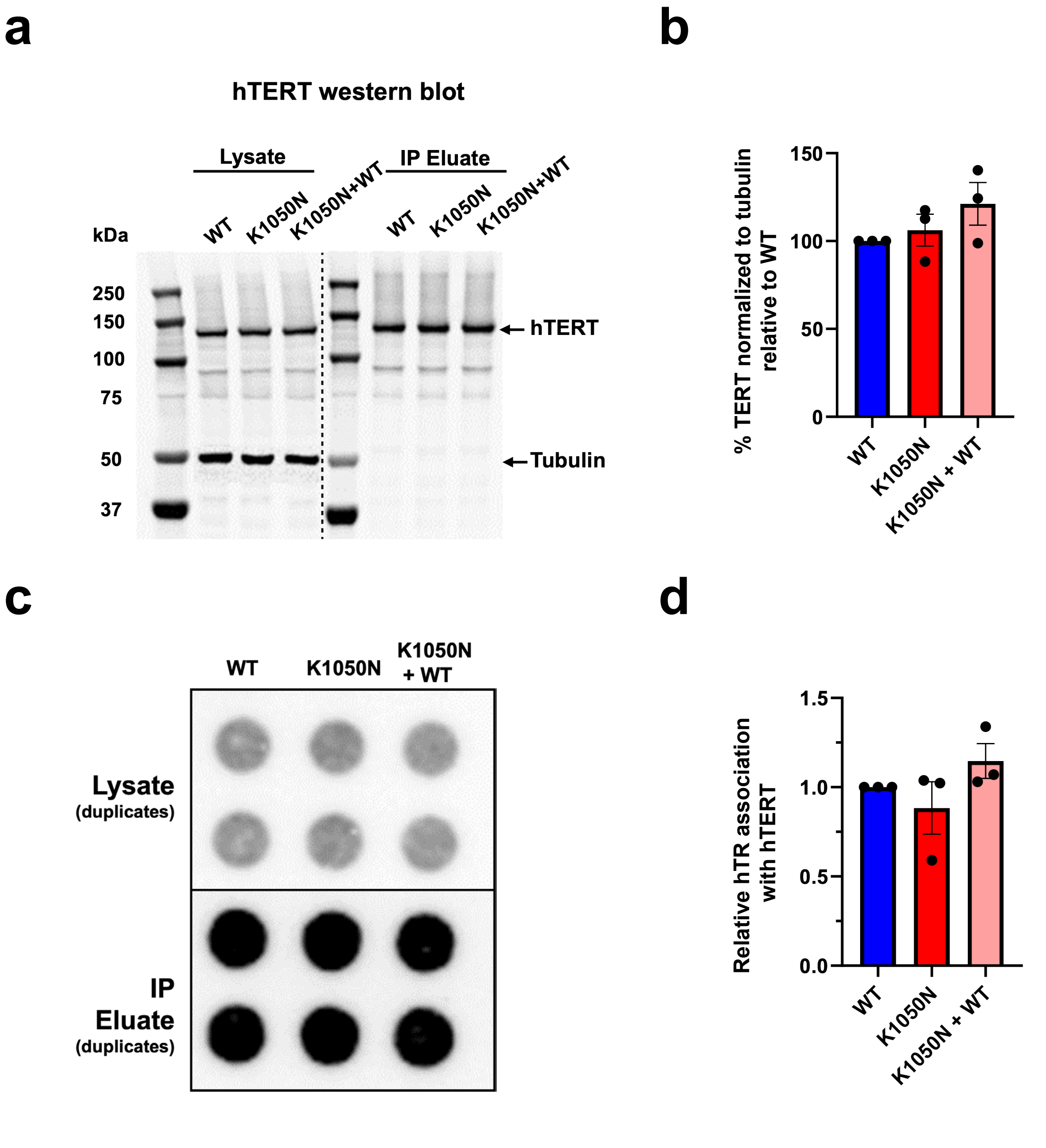

Supplementary Figure 2. TERT K1050N does not impact hTERT expression or stability, or hTR association with hTERT. a) Western blot of WT, K1050N and K1050N + WT from equal volumes of cell lysate of transfected HEK293T cells (Lysate) and equal volumes of immunopurified FLAG-tagged hTERT (IP Eluate), probed using anti-FLAG and anti-tubulin antibodies. IP Eluate lanes show successful purification of hTERT. Dotted line represents removal of lanes of the gel containing replicates of the same experiment. **b)** Densitometry measurements of hTERT expression, normalized to tubulin expression in transfected HEK293T cell lysate (lysate lanes). Error bars represent mean + SEM (n=3). **c)** Northern dot-blot showing relative amounts of hTR in equal volumes of cell lysate of transfected HEK293T cells (Lysate) and hTR associated with hTERT in immunopurified FLAG-tagged hTERT (IP Eluate; equal volumes loaded). **d)** Relative number of hTR molecules associated with each molecule of hTERT compared to WT, based on relative recovery of hTR and hTERT following immunopurification, normalized to the relative amount of hTR in cell lysates. Error bars represent mean + SEM (n=3). Data points represent independent immunopurified telomerase samples.

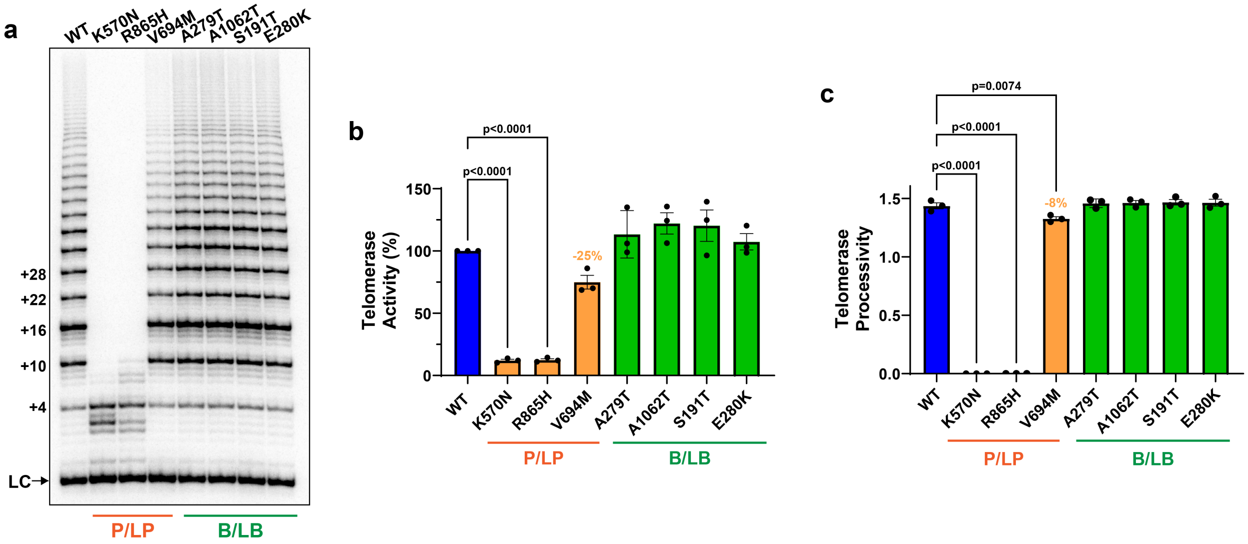

Supplementary Figure 3. Impact of seven different *TERT* variants (control panel) on telomerase activity and processivity. a) Direct telomerase assay of immunopurified telomerase containing known P/LP or B/LB variants as controls. Each prominent band represents the addition of a TTAGGG repeat to the DNA substrate, with the number of nucleotides added indicated on the left. LC: labelled, biotinylated 30nt oligonucleotide recovery and loading control. **b)** Specific activity of the panel of control variants compared with WT telomerase. **c)** Processivity of the panel of control variants relative to WT telomerase. Processivity is defined as the average number of DNA repeats synthesized before enzyme dissociation from DNA. Data points represent independent immunopurified telomerase samples, with error bars indicating mean + SEM (n=3). Abbreviations: WT, wild-type; P/LP, pathogenic or likely pathogenic; B/LB, benign or likely benign. Plots created with GraphPad Prism version 10.0.0 (www.graphpad.com).

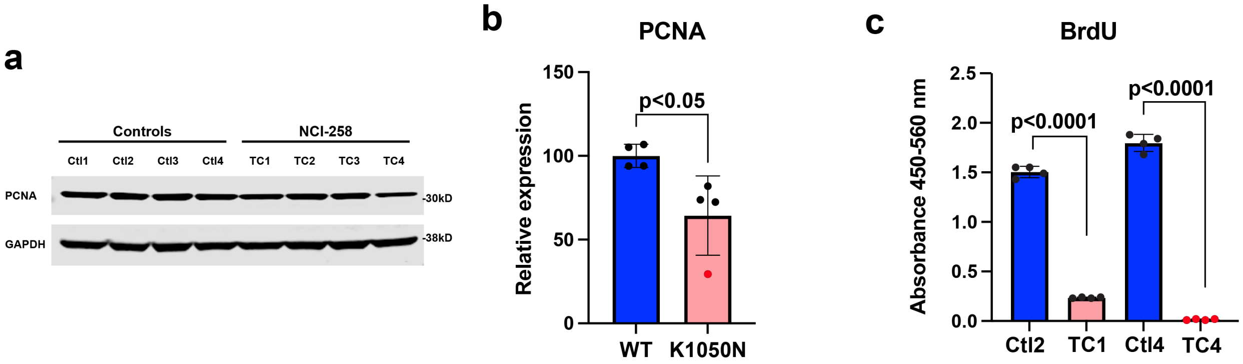

Supplementary Figure 4. **The *TERT* p.K1050N variant reduces cell proliferation in primary patient-derived fibroblasts. a)** Western blot showing the protein expression of PCNA. GAPDH was used as a loading control. **b)** Quantification of western blots. PCNA protein levels were normalized to GAPDH expression and calculated as a percentage of controls. **c)** BrdU cell proliferation assay revealing the decreased BrdU incorporation in carrier fibroblasts compared with their matched controls, indicating reduced cell proliferation. The red dot represents the p.K1050N homozygous sample (TC4). The bars represent the mean absorbance ± standard deviation (n=4). Statistical significance was determined by Student's t-test. Abbreviations: Ctl, control; WT, wild-type; TC1-3, *TERT* c.3150G>C p.K1050N heterozygotes, TC4, *TERT* c.3150G>C p.K1050N homozygote. Plots created with GraphPad Prism version 10.0.0 (www.graphpad.com).

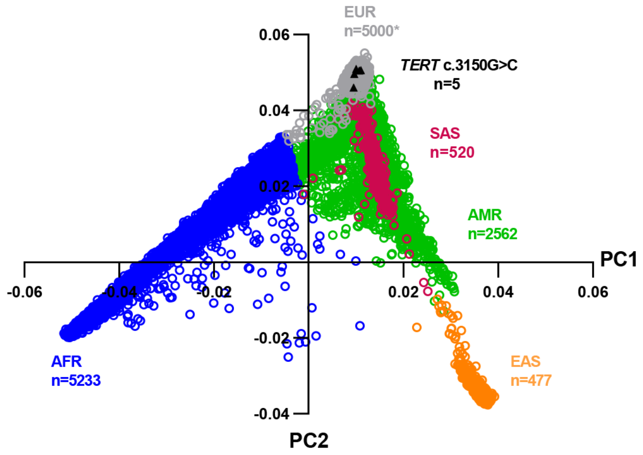

Supplementary Figure 5. Principal Component Analysis (PCA) plot illustrating genetic ancestry of the p.K1050N heterozygotes from Geisinger. Major continental populations are represented as follows: African (AFR, blue), East Asian (EAS, orange), Admixed American (AMR, green), South Asian (SAS,red), and European (EUR, gray). Each ring represents an individual, and the color corresponds to their respective population group as inferred from reference panels. The *TERT* c.3150G>C heterozygotes (black triangles) cluster closely with European (EUR) individuals, indicating their genetic similarity to this population group. *For illustration purposes and to ensure clear separation between populations, the number of EUR individuals was limited to 5000. Principal components 1 and 2, which explain the majority of the genetic variation, are plotted on the x- and y-axes, respectively. Plots created with GraphPad Prism version 8.1.2 (www.graphpad.com).

Uncropped gel corresponding to Figure 3a

Green box:
Lanes as labelled in Fig 3a

Red box (not in figure):
replicate of same experiment with an extra WT lane

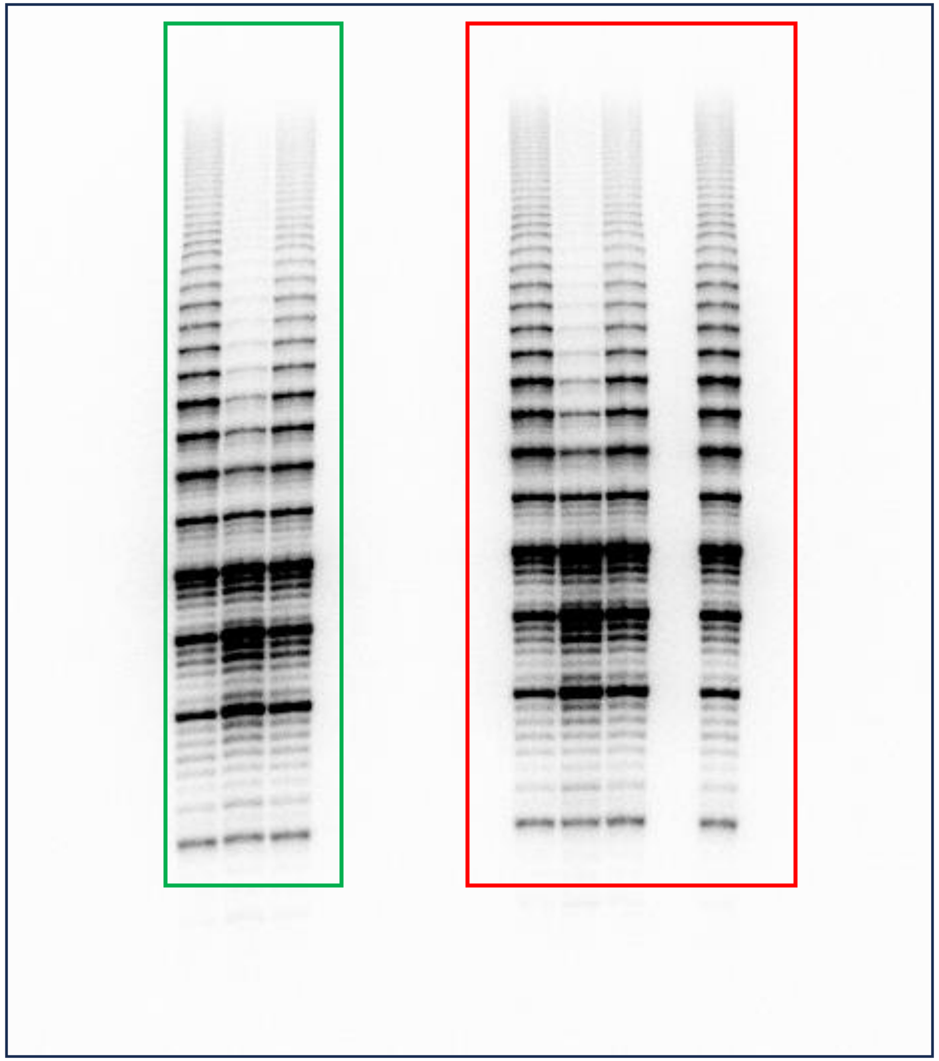

Unprocessed, uncropped gel corresponding to Supplementary Figure 2a

Blue box:
2 separate replicates of cell lysates of WT, K1050N and K1050N + WT hTERT transfections

Purple box:
2 separate replicates of immunoprecipated eluates of WT, K1050N and K1050N + WT hTERT transfections

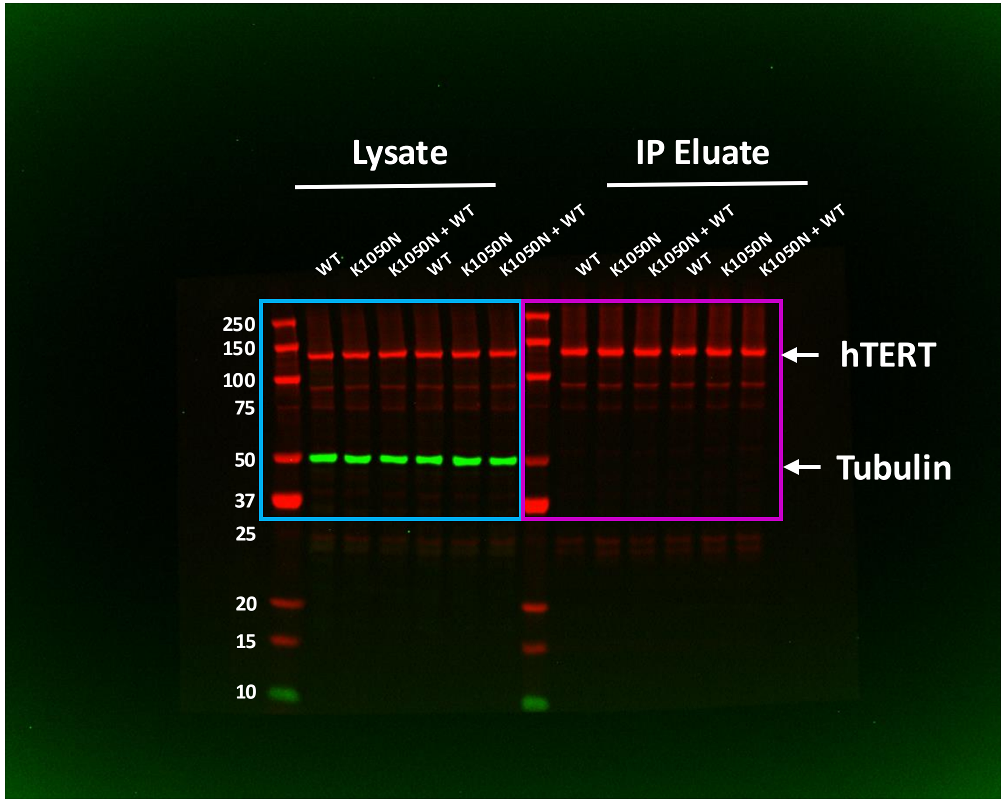

Uncropped blot corresponding to Supplementary Figure 2c

Green boxes:
Samples as labelled in Supp. Fig. 2c

Blue boxes (not in figure):
Replicate of same experiment

Purple boxes:
Erroneously loaded samples (not included in quantitation)

Red box (not in figure):
Unrelated irrelevant experiment

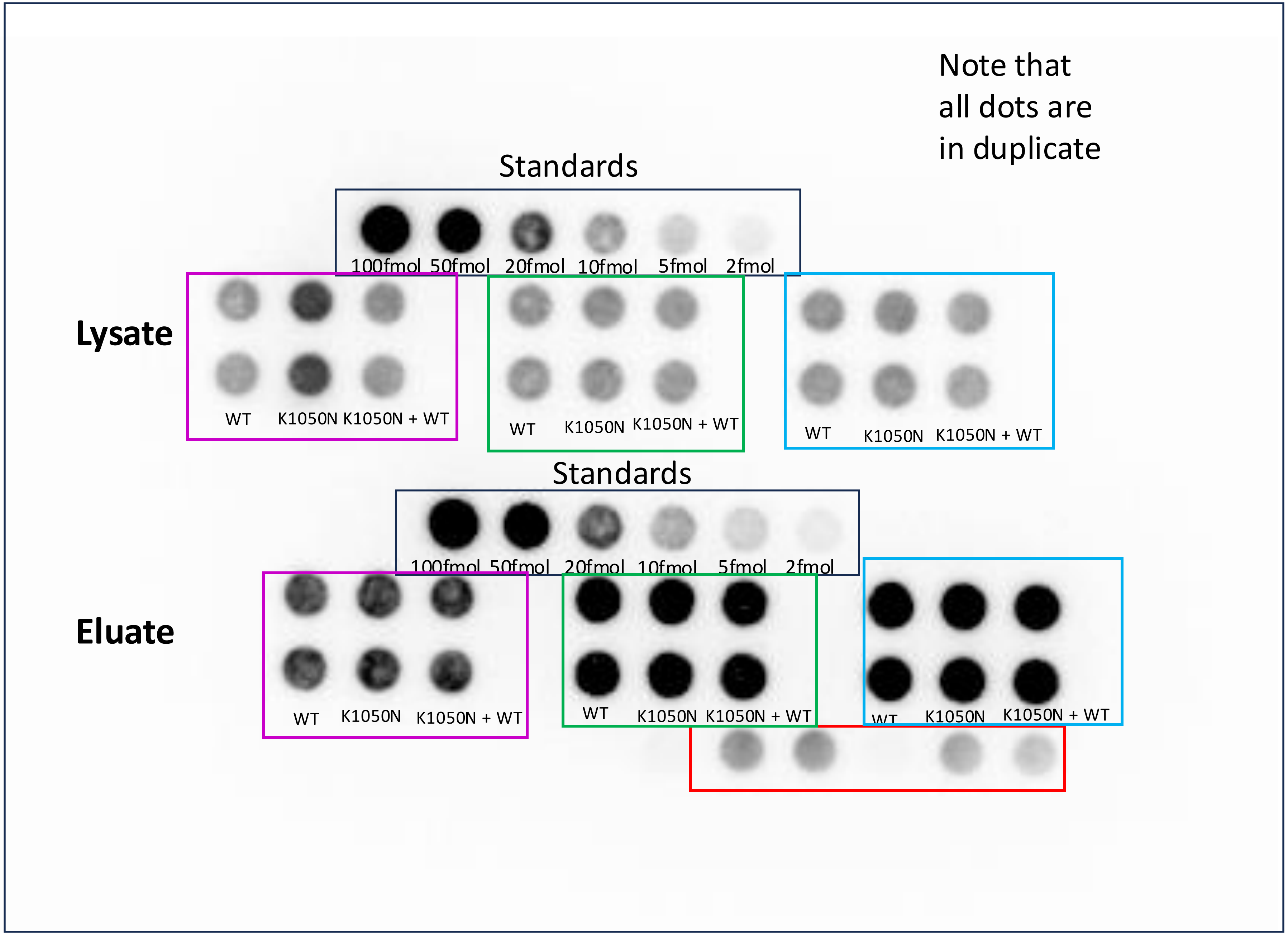

Uncropped gel corresponding to Supplementary Figure 3a

Green box:
Lanes as labelled in Supp. Fig 3a

Red box (not in figure):
Unrelated irrelevant experiment

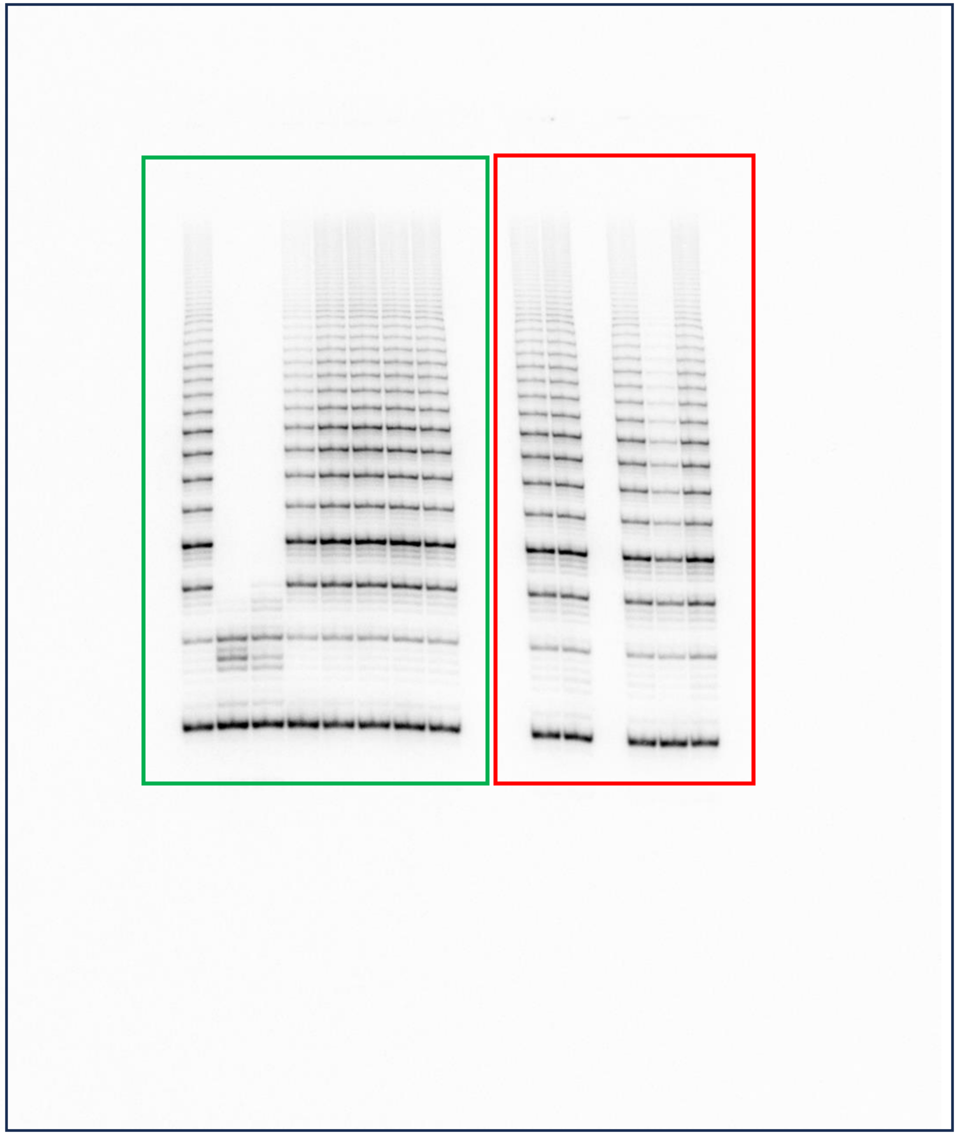

Unprocessed, uncropped blot corresponding to Supplementary Figure 4a

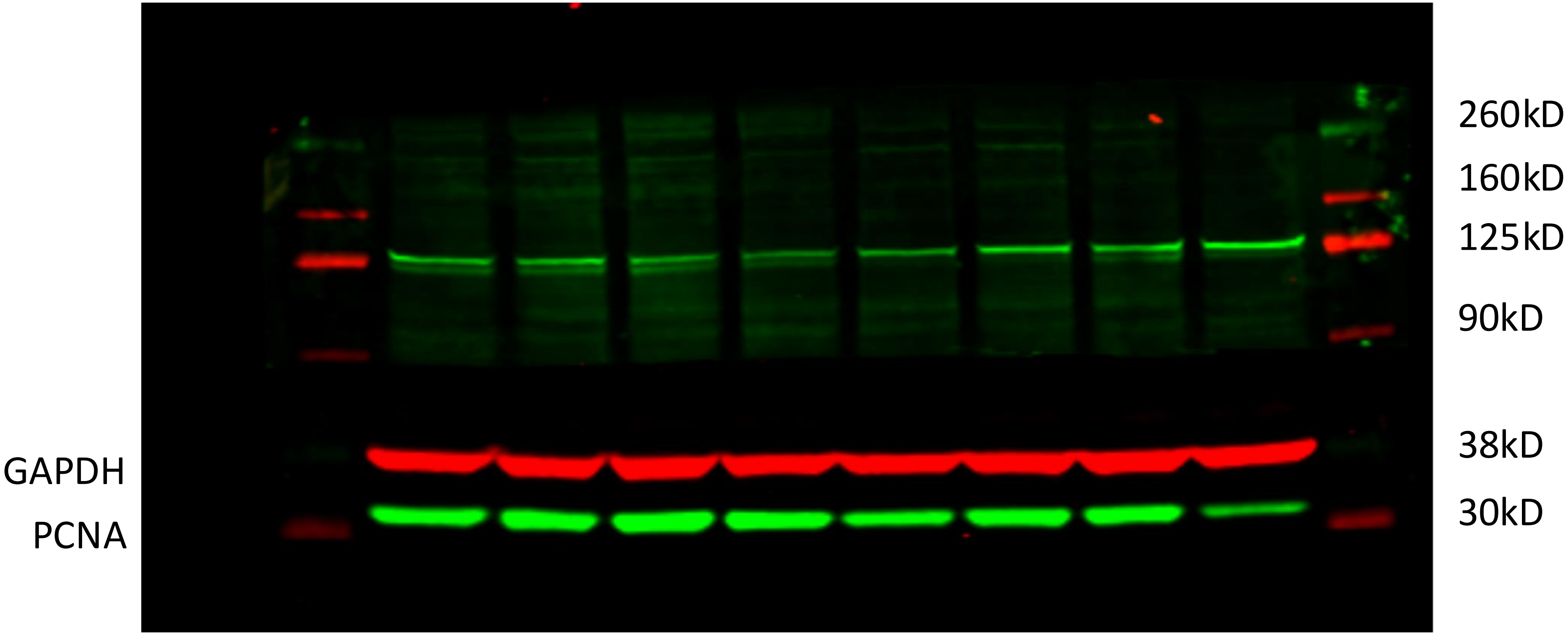

Supplement: Supplementary file 1 — Supplementary materials [file 41525_2025_501_MOESM1_ESM.pdf]
